# Implementing ePROM in specialist palliative home care: the professionals’ perspective – a mixed-methods study

**DOI:** 10.1177/26323524231186827

**Published:** 2023-08-06

**Authors:** Isabel Burner-Fritsch, Stefanie Kolmhuber, Farina Hodiamont, Claudia Bausewein, Katerina Hriskova

**Affiliations:** Department of Palliative Medicine, University Hospital, LMU Munich, Bahnhofstraße 20, 83673 Bichl, Germany; Department of Palliative Medicine, University Hospital, LMU Munich, Munich, Germany; Department of Palliative Medicine, University Hospital, LMU Munich, Munich, Germany; Department of Palliative Medicine, University Hospital, LMU Munich, Munich, Germany; Department of Palliative Medicine, University Hospital, LMU Munich, Munich, Germany

**Keywords:** electronic patient-reported outcome measures, palliative care, palliative home care, patient-reported outcome measurement, professionals’ perspective

## Abstract

**Background:**

Over the last decades, patient-reported outcome measures (PROM) have been developed for a better understanding of patient needs. The Integrated Palliative Care Outcome Scale (IPOS) is an internationally recommended PROM in palliative care. The validated electronic version of IPOS (eIPOS) was implemented in four German specialist palliative home care (SPHC) teams for use in everyday clinical practice. Patients reported symptoms and concerns via eIPOS, which were transmitted directly to the electronic patient record of the respective SPHC team.

**Objectives:**

The aim of the study was to describe and explore the health care professionals’ (HCPs’) experiences regarding acceptance and use of eIPOS in clinical practice in SPHC.

**Design:**

The mixed-methods sequential explanatory design comprised an anonymized quantitative online survey followed by qualitative focus groups.

**Methods:**

The online survey asked in both closed and open questions for HCP’s experience with eIPOS. Ambiguous results from the survey were discussed in two focus groups. Survey data were analysed with descriptive and univariable statistics, and the framework approach was used for qualitative data. In a further step, we conducted integrated analysis of quantitative and qualitative results using joint displays.

**Results:**

All HCPs of the four SPHC teams (*n* = 52) were invited to participate. HCPs participating in the survey (*n* = 32) and the focus groups (*n* = 7) saw potentials for implementing ePROM in palliative home care – as far as it is technically easy to handle and can be easily integrated into clinical practice.

**Conclusion:**

Successful use of ePROMs is affected by the possibility of easy integration into the teams’ different structures and processes and the HCPs’ perceptions of potentials regarding ePROM use in SPHC.

**Registration:**

The study is registered on clinicaltrials.org (NCT03879668).

## Plain Language Summary

The use of electronic patient-reported outcome measures in specialist palliative home care: what do professionals think about it? A mixed-methods study

Patient-reported outcome measures (PROMs) are short questionnaires developed to assess a patient’s health status at a particular point in time. The Integrated Palliative Care Outcome Scale (IPOS) is such a questionnaire, and eIPOS is an electronic version of IPOS. IPOS asks about patients’ symptoms and problems when they suffer from advanced diseases. We conducted this study to understand what health care professionals (HCPs) think about electronic PROMs (ePROMs) in palliative home care. We first asked the HCPs to answer questions in an online survey. Then, HCPs discussed the use of eIPOS in small discussion groups. This study design is called ‘Mixed-Methods sequential design’. We found that all HCPs used the information they received through eIPOS – some frequently and some less often. Many HCPs see potential in using ePROMs to support care. For example, because ePROMs help them to understand patients’ symptoms and problems better. However, they also pointed out that eIPOS needs technical improvement. Also, the procedures of eIPOS need to fit into the work routine of the palliative care team. The findings demonstrate the perspectives of HCPs on ePROM. These are valuable to understand how ePROM can be implemented in palliative home care. We can also learn about how to implement other digital tools in other settings of palliative care.

## Introduction

Health systems around the world are facing major challenges as the number of older people with multi-morbid conditions increases and the need for palliative care will rise.^1^ To address the current challenges, variations in the quality of health care need to be approached by improving outcomes.^2^ The best way to achieve this is to measure individual patient-centred outcomes.^3^ Patient-reported outcome measures (PROMs) are validated questionnaires completed by patients to measure their perceptions of their own health status/well-being.^4^ With the increasing adaptation of internet-enabled devices in our everyday life, electronic PROMs (ePROMs) appear as a feasible option to improve the quality of assessment and could play an important role in the development of new digital health interventions.^5^ Electronic as well as classic PROMs can be used at a single point of time to support multi-perspective assessment or regularly, to measure the effectiveness of care interventions or monitor health status. Use in palliative care settings shows that (e)PROMs can potentially foster person-centredness, patient empowerment, better communication and support identification of not recognized symptoms.^6–9^ Exemplary instruments developed especially for palliative care include the Edmonton Symptom Assessment Scale,^10^ the European Organization for Research and Treatment of Cancer Quality of Life Questionnaire Core 15 Palliative^11^ or the Palliative Care Outcome Scale (POS).^12^ The Integrated POS (IPOS), as a further development of the POS, is a widely recommended PROM in palliative care and validated in many languages as well as diverse palliative care setting.^13–16^ When palliative care patients are no longer able to provide information about their palliative care needs, IPOS can be also used as proxy tool by professionals. It covers patients’ main concerns, common symptom burden, patient/family distress, existential well-being, sharing feelings with family or friends, information received and practical concerns, in 17 items within a timeframe of 3 or 7 days.^14^

Given the afore-mentioned benefits of (e)PROM use, we conducted the project Palli-MONTOR (‘Monitoring of palliative care needs in specialist home-based palliative care using an electronic version of the Integrated Palliative Care Outcome Scale’, clinical trials NCT03879668), which aims to test the electronic version of the previously validated paper-based IPOS (eIPOS) in a German specialist palliative home care (SPHC) setting.^17,18^ Multi-professional teams are typical for SPHC which provide end-of-life care for patients with complex symptom burden using a holistic and patient-centred approach.^19^ Implementation means the systematic introduction of an innovation, using a planned process with the goal of integration into in daily care routine.^20^

Despite potential benefits, implementing innovations such as ePROMs holds various challenges. Dealing with stakeholders’ resistance is one great potential barrier for the success of implementing change.^21^ Thus, health care professionals (HCPs) and patients are important factors for the success of the implementation process in health care. Therefore, our study aimed to describe and explore the HCPs’ experiences of using the eIPOS in everyday clinical practice in SPHC. The objectives of the study were: (i) the effort of eIPOS use, (ii) its implications on care as well as (iii) developed routines in clinical practice.

## Materials and methods

### Study design

**Figure 1.**
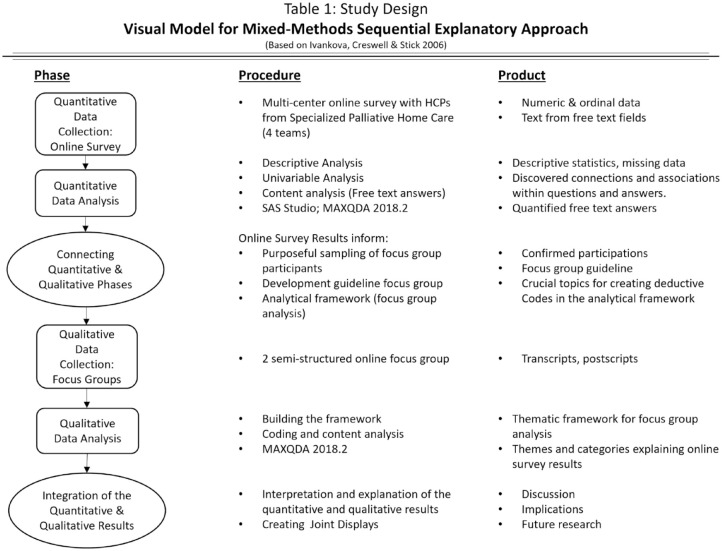
Study design: visual model for mixed-methods sequential explanatory approach (based on Ivankova, et al. 2006).^25^

Based on the taxonomy of Creswell and Plano Clark, a mixed-methods sequential explanatory design was chosen to gain deeper understanding of HCPs’ perspective.^22^ This mixed-methods study followed the guidelines for the design, implementation and reporting of findings of the good reporting of a mixed-methods study (GRAMMS).^23^ First, a specially developed and anonymized online survey was addressed to all HCPs of the four SPHC teams participating in the overall project (*n* = 52). To help interpret ambiguous results of the online survey, they were discussed with HCPs from the four participating SPHC teams in two online focus groups via zoom (see Figure 1). The details about the overall project ‘Palli-MONITOR’ are described elsewhere.^18^ The overall project consists of phase I (development) and II (feasibility) following the Medical Research Council framework for complex interventions,^24^ and the reported study was part of the feasibility phase. Briefly, the eIPOS was implemented in four SPHC teams without experience in PROM for use in clinical routine. Patients cared for by the teams reported their symptom burden and concerns via eIPOS. Values completed online were transmitted directly into the electronic patient record of the responsible SPHC team, and professionals were required to view the transmitted values before the next planed patient contact.

### Setting and participants

Participating teams were recruited in scope of the overall study Palli-MONITOR. As SPHC provides holistic end-of-life care with symptom control and support regarding psychological, social or spiritual issues, multi-professional teams consist of nurses and doctors and partly additional professions like social workers, psychologists or physiotherapists. All provide care for adult patients with complex symptom burden, suffering from life limiting oncological or non-oncological disease. Apart from this, structure and organization of the participating teams differ widely. Two teams are located in rural and two in urban regions of Bavaria, Germany. The participating teams are working with two different software (SW) systems for documentation and administration, which offer the same functions but differ in design and workflows. For example, the button indicating that a patient usually uses eIPOS is only visible after opening the patient’s individual case report in SW1, while SW2 makes this button visible in the overview of all patients. Access to the eIPOS report sent by the patients is similar: In both software programmes, the individual case report must be opened to see the values from eIPOS. The urban teams participating in the study are using SW1, whereas the rural teams use SW2.

In both online-survey and focus groups, participants were informed about the respective parts of the study and the option to drop out at any time. Informed consent was provided via a dialog box at the beginning of the survey and with signed consent declaration of focus groups‘ participants, respectively. The study population included all 52 HPCs (physicians and nurses) of the SPHC teams, being actively involved in the use of eIPOS. All were invited to participate in the online survey. As the objective of the study was understanding professionals’ experience using eIPOS in SPHC as part of the overall study Palli-MONITOR, the basic sample was determined by the number of professionals working in the respective teams. Due to the small sample, we did not collect any information about gender, age, profession and experience to ensure anonymity in the small and highly connected German SPHC setting. Purposeful sampling for the focus groups was informed by the results of the online survey. To foster multi-perspectives in the discussion, we aimed for a diverse group composition considering the following criteria: region (urban/rural), used IT-system (SW1/SW2) and profession (nurse/physician). It was possible for HCPs to participate in both the survey and the focus groups.

### Data collection

Data collection took place in April and May 2021.

#### Online Survey

All physicians and nurses of the four SPHC teams were invited via email to participate. The questionnaire was based on a survey used in a similar project in Freiburg, Germany.^26^ Our questions focussed on the following topics, as their importance is highlighted by relevant literature: the effort of use, the ability to integrate the system and its role in daily care routine.^20,27–29^ The survey contained closed questions asking for the HCPs’ experiences with eIPOS in routine care and open questions to report barriers and suggestions for improvement. The survey questions are summarized in Table 1. The completion time was estimated to be about 7 min. Participants were able to skip questions and complete the survey in one or more sessions. After cognitive testing, small adjustments were made before the start of the survey. The survey was open for 4 weeks. After 2 weeks, the HCPs received an email as reminder.

**Table 1.** Descriptive statistics.

Questions	Answers	*n* = 32	%	
To what extent do you agree with the following statements regarding the patients’ recording of symptoms and palliative care needs using eIPOS?				
Looked at the patients’ statements	*Never*	0		0
	*Seldom*	7		21.9
	*Sometimes*	8		25.0
	*Often*	13		40.6
	*Always*	4		12.5
Discussed the patients’ statements in the team	*Never*	0		0
	*Seldom*	6		18.8
	*Sometimes*	16		50.0
	*Often*	7		21.9
	*Always*	3		9.4
Better identification of patients’ symptoms and palliative needs by using eIPOS	*Never*	1		3.1
	*Seldom*	13		40.6
	*Sometimes*	11		34.4
	*Often*	7		21.9
	*Always*	0		0
Better identification of patients’ burden by using eIPOS	*Never*	1		3.1
	*Seldom*	8		25.0
	*Sometimes*	15		46.9
	*Often*	8		25.0
	*Always*	0		0
Adaption of care based on patients’ responses	*Never*	9		28.1
	*Seldom*	8		25.0
	*Sometimes*	9		28.1
	*Often*	6		18.8
	*Always*	0		0
Information provided by the patients was useful for HCPs’ work	*Never*	4		12.5
	*Seldom*	7		21.9
	*Sometimes*	10		31.3
	*Often*	8		25.0
	*Always*	3		9.4
Information provided by the patients as an opportunity to address certain topics with patients	*Never*	6		18.8
	*Seldom*	9		28.1
	*Sometimes*	11		34.4
	*Often*	6		18.8
	*Always*	0		0
Patients’ statements as an opportunity to discuss the patients’ stresses with colleagues	*Never*	5		15.6
	*Seldom*	11		34.4
	*Sometimes*	8		25.0
	*Often*	7		21.9
	*Always*	1		3.1
Have you noticed any changes as a result of using eIPOS (in clinical practice)?				
Changes in treatment of physical stress/symptoms	*Worsening*	1	3.1	
	*No change*	17	53.1	
	*Improvement*	13	40.6	
	*I don’t know*	1	3.1	
Changes in counselling for social problems	*Worsening*	0	0	
	*No change*	16	50.0	
	*Improvement*	10	31.3	
	*I don’t know*	6	18.8	
Changes in patients’ quality of life	*Worsening*	0	0	
	*No change*	14	43.8	
	*Improvement*	14	43.8	
	*I don’t know*	4	12.5	
Changes in doctor–patient communication	*Worsening*	0	0	
	*No change*	13	40.6	
	*Improvement*	16	50.0	
	*I don’t know*	3	9.4	
Changes in communication about patients’ burdens in the team	*Worsening*	0	0	
	*No change*	14	43.8	
	*Improvement*	14	43.8	
	*I don`t know*	4	12.5	
To what extent do you agree with the following statements regarding the integration of *electronic* recording of patients’ symptoms and palliative care needs into clinical practice?				
Successfully developed routines for the use of the patients’ data	*Agree*	7	21.9	
	*Neutral*	10	31.3	
	*Disagree*	15	46.9	
Effort of using the patients’ data is appropriate with the benefit	*Agree*	6	18.8	
	*Neutral*	11	34.4	
	*Disagree*	15	46.9	
Display of the eIPOS in the documentation system allows easy inclusion of patients’ information	*Agree*	14	43.8	
	*Neutral*	5	15.6	
	*Disagree*	13	40.6	
How do you estimate the effort of using eIPOS for . . .				
HCPs	*Particularly low*	3	9.4	
	*Low*	22	68.8	
	*High*	5	15.6	
	*Particularly high*	0	0	
	*I don’t know*	2	6.3	
Would you support further use of eIPOS after the project period?				
	*No*	13	40.6	
	*Yes, without changes*	7	21.9	
	*Yes, with changes (free-text)*:	10	31.3	
	*Implementation*	2		
	*Technology*	5		
	*Setting*	3		
	*Missing*	2	6.3	
What suggestions do you have for improving the electronic recording of patients’ symptoms and palliative care needs? (multiple answers are possible)				
	*Implementation*	3	9.4	
	*Technology*	7	21.9	
	*Setting*	3	9.4	
	*None*	3	9.4	
	*PROM*	3	9.4	
	*Others*	1	3.1	
	*Not reported*	16	50.0	
What barriers did you perceive during the project? (multiple answers are possible)				
	*Implementation and study conditions*	11	34.4	
	*Technology*	6	18.8	
	*Setting*	14	43.8	
	*None*	2	6.3	
	*COVID-19*	1	3.1	
	*Others*	1	3.1	
	*Not reported*	7	21.9	
Which documentation system do you use?				
	*Software 1*	12	37.5	
	*Software 2*	19	59.4	
	*Missing*	1	3.1	

#### Focus groups

Subsequently, we conducted semi-structured online focus groups to explore contrasting experiences of the participants. To counter recruitment problems, we offered two focus groups at different times of the day. The interview guide was informed by results of the online survey and covered the following topics: attitude towards eIPOS (personal and in the team), use of the eIPOS in daily care in the SPHC setting technical implementation. Two researchers moderated the groups (IBF and KH), and one researcher (SK) provided technical support during the discussion. The conversations were audio recorded and transcribed verbatim; postscripts saved information that were not captured in the audios. To ensure confidentiality, all data were anonymized.

### Analysis

Data were analysed in three phases: descriptive analysis of the online survey, qualitative analysis of transcripts and postscripts of the focus groups and an integrated analysis of both quantitative and qualitative data.

#### Online Survey

The closed survey questions were analysed descriptively, and the absolute and relative frequencies were reported. The Chi-square test and the Fischer exact test were used to test for statistically significant differences between categorical variables. Due to the small sample size, only the Fischer exact test was reported. The objective of the univariable tests was to examine the dependence of the effort of use, frequency of use, perceived changes and software type on other factors identified in the survey. Univariable analysis was conducted with SAS Studio (SAS 9.04.01M6P110718). Qualitative content analysis was performed to examine the free-text answers, using MAXQDAv.2018.2.^30^ Analytic consensus was reached through coding review by the research team (IBF, SK and KH).

#### Focus Groups

To analyse transcripts and postscripts, we followed the framework approach using MAXQDAv.2018.2. The framework approach developed by Ritchie and Lewis allows transparent and structured management and analysis of qualitative data.^31^ After getting familiar with the data material and identifying important topics, the content is displayed in thematic charts that allow further analysis and interpretation.^32^ Our thematic framework was built with both deductive codes derived from the results of the online survey and inductive codes to cover all aspects of the data. Coding reviews and discussion of disagreements in the team (IBF, SK and KH) supported consistent analysis.

#### Integration

For the integrated analysis of both data sets, we developed joint displays.^22^ These combine the quantitative detailed results with thematically matching qualitative data. The goal of the triangulation was to elucidate the survey outcomes with our qualitative findings.

## Results

The overall response rate in the online survey was 62% (32/52). Nineteen out of 32 participants (59%) used software SW2, and 12/32 (38%) participants used software SW1. One participant did not answer this question. One of the nine questions, which asked whether the IPOS could be considered a suitable basis for an ePROM, was misinterpreted by most participants. The answers referred to the implemented electronic IPOS instead of the IPOS as a suitable digital PROM instrument. Therefore, it was not included in the analysis. For an overview of descriptive survey results, see Table 1. Table 2 shows the results of the univariable analysis. In the two focus groups (FG1 *n* = 3 and FG2 *n* = 4), four participants used SW2 and three participants used SW1.

**Table 2.** Univariable analysis.

	Effort of use for HCPs		OR [95% CI]	*p* Value
	Low, *n* (%)	High, *n* (%)		
Information provided by eIPOS perceived as useful:			16 [1.09; 234.25]	0.06
Seldom, sometimes, often, always	24 (80.0%)	3 (10.0%)		
Never (ref.)	1 (3.3%)	2 (6.7%)		
	Software type		OR [95%-CI]	*p* Value
	SW1, *n* (%)	SW2, *n* (%)		
Effort of using the patients’ data perceived as commensurate with the benefit			12.86 [1.27; 130.54]	0.02
Agree	5 (16.1%)	1 (3.2%)		
Neutral/disagree (ref.)	7 (22.6%)	18 (58.1%)		
Wish for further use of eIPOS			1.78 [0.38; 8.23]	0.70
Yes	8 (27.6%)	9 (31.0%)		
No (ref.)	4 (13.8%)	8 (27.6%)		
Effort of use for HCPs			0.84 [0.12; 6.03]	1.00
Low	9 (30.0%)	16 (53.3%)		
High (ref.)	2 (6.7%)	3 (10.0%)		
Display of eIPOS in software allows easy integration of patients’ information			0.24 [0.05; 1.19]	0.14
Agree	3 (9.7%)	11 (35.5%)		
Neutral/disagree (ref.)	9 (29.03%)	8 (25.8%)		
	Opened eIPOS		OR [95%-CI]	*p* Value
	always/often, *n* (%)	sometimes/seldom, *n* (%)		
Successfully developed routines for the use of the patients’ data			4.80 [1.07; 21.45]	0.07
Agree/neutral	12 (37.5%)	5 (15.6%)		
Disagree (ref.)	5 (15.6%)	10 (31.3%)		

CI, confidence interval; OR, odds ratio.

ref. indicates the reference categories.

### eIPOS as support tool in everyday care

All participants had opened eIPOS and looked at the patients’ statements during the project at least once (*n* = 32), more than 50% even often or always (*n* = 17). Furthermore, all HCPs had discussed patients’ statements submitted via eIPOS in the team, about one-third even often or always (*n* = 10). Participants who opened eIPOS regularly (always/often) had a 43 times higher chance to discuss patients’ statements in the team (*p* = 0.0003). In the focus group, it was mentioned that differences between patients’ statements and HCPs’ assessment were a good starting point for discussion in the team. Nearly all HCPs (*n* = 31, 97%) experienced a better identification of patients’ burden or symptoms and palliative care needs in the study period at least once. However, 23/32 (72%) stated that this happened only *seldom* or *sometimes* regarding the patients’ burden, and 24/32 (75%) saw only *seldom* or *sometimes* better identification of symptoms and care needs.

In the discussion, professionals claimed that the free-text questions of eIPOS are of special interest for identification of unrecognized aspects. Benefits using eIPOS were mainly perceived in the identification of psychosocial issues. A total of 28 participants (88%) stated that the information sent via eIPOS was useful for their work and 23 (72%) adapted care. One HCP explained in which way he used the provided information: *“Is it at the computer in the morning for team coordination. And seeing [. . .] that someone has clicked a three or a four, simply gave me a hint that we have to be active today”* (HCP, SW2 user). The large majority of the HCPs (*n* = 26, 81%) perceived no effect of eIPOS on their relationship with the patients. Nevertheless, a focus group participant voiced concerns that the relationship with patients could be negatively affected in case eIPOS-reported symptom burden might not be followed by reaction from the team because the patients’ statements are not noted timely. For most participants in the survey, the information provided by the patients can be seen as an opportunity to address certain topics with the patients (*n* = 26, 81%) or to discuss with colleagues (*n* = 27, 84%), even though many stated that this was only *seldom* the case (address topics with patients: *n* = 9, 28%; discuss with colleagues: *n* = 11, 34%). One HCP described that eIPOS revealed differences between patients’ views on symptom burden and the HCPs’ assessment, what was a particular impetus for in-team discussions.

### eIPOS use: implications on care

Leaving *I don’t know*-answers aside, 13 participants perceived an improvement in the treatment of physical symptoms, while 17 HCPs did not notice any change. Regarding psychosocial aspects of care, no change was perceived by 13 participants in the treatment of mental distress, whereas slightly more HCPs noticed an improvement (*n* = 15). Half of the HCPs noted an improvement in the patients’ quality of life (*n* = 14). Mostly no change was perceived in counselling for social problems (*n* = 16), accompanying during existential crises (*n* = 18) or in spiritual concerns (*n* = 29). Regarding communication, 16 HCPs perceived improvement in the exchange with patients and 14 participants noticed positive effects on the intra-team communication. In the focus group, one HCP explained that psychological issues often suffer in case of hectic workflow. Focusing on communication about crucial aspects, speaking about spiritual issues might be neglected: *“So for me, it would be, if then, these free fields and these psycho-social and emotional issues. Were you at peace with yourself? After all that’s a nice question, [usually] I don’t ask it like that”* (HCP, SW2 user). Professionals assumed that for some patients it might be easier to mention psychological issues, typing them in eIPOS.

### Implementing eIPOS in daily care routine

Nearly half of the survey participants (*n* = 15, 47%) expressed that they had not developed routines for using patients’ information from eIPOS in clinical practice. Focus group results revealed that the digital information display did not meet the needs, especially regarding SW1. Because an active ‘search’ for newly transmitted values was required, eIPOS was often not opened until the documentation was entered after a patient contact. However, seven professionals (22%) reported successful integration into the individual clinical practice. In one of the focus groups, an SW2 user discussed with an SW1 user how their teams are dealing with the newly arriving eIPOS. The SW2 user described the clinical care routine in her team with a person who coordinates patients to the staff in the morning: *“If I as a staff member get five patients, then I have to check the documentation of each patient in our documentation system in the morning. And … then I also open eIPOS. Just like I read my colleagues’ documentation from before to get an overview again. Because you’re not at work every day … because maybe yesterday a colleague was taking care of this patient”.*

This seems to be different in SW1 users’ team, where HCPs do not share patients among each other and are very involved in their current situation. Therefore, in this team, HCPs do not check the documentation every day. Another focus group participant from the second team using SW1 confirms this practice for his own team. The conclusion from this dialogue between focus group participants about the different routines with eIPOS in SPHC is reflected by SW2 user: *“Ah, ok! That’s the reason, it just depends on the way the work is done!”*. Interestingly, the second team using SW2 was unable to develop routines for using eIPOS information in daily practice but did not indicate workflow as the reason. Rather, it was due to the patient population with a high complex symptom burden and very short treatment duration, as reported by one focus group participant. Only a few patients in this team were able to use eIPOS. However, univariable analysis showed that HCPs who opened eIPOS regularly (always/often) had a 12 times higher chance to report successfully developed routines for the use of eIPOS (*p* = 0.06).

### Effort of using eIPOS

The majority (*n* = 25, 78%) assessed the effort of using eIPOS as low. However, in the focus groups, at least one HCP using SW1 perceived the efforts of integrating eIPOS as too high. Furthermore, the effort of using the patients’ data was perceived for nearly half of the professionals as not appropriate with the benefit (*n* = 15, 47%), though for six participants (19%), the effort–benefit relation was good. Univariable analysis showed statistical significance with the used software: HCPs who used SW1 had a nearly 13 times higher chance of perceiving the effort–benefit ratio as appropriate (*p* = 0.02). The assessment regarding the display of eIPOS was divided: 14 participants (44%) agreed that the readout in the documentation system allows easy inclusion of patients’ information; however, 13 HCPs (41%) stated the opposite. This difference was not statistically significant (*p* = 0.14). The discussion in the focus groups unveiled relevant differences between the two software systems. In SW2, all patients using eIPOS were labelled by a button in the patient overview. Clicking on it, the HCP could easily see transmitted values: *“When I open it, I see the button: that’s a patient who is taking part in the study. And I can click on it and see it [values transmitted *via* eIPOS] right away”* (HCP, SW2 user). In contrast, in SW1, the team needed to select the particular patient, before a button indicating eIPOS use appeared. A discussion between two HCPs indicated that differences in software design were not the main reason for the varied perception of effort. They made clear that divergent organizational structure of clinical practice is of great impact as well.

### Suggestions for improvement

**Figure 2.**
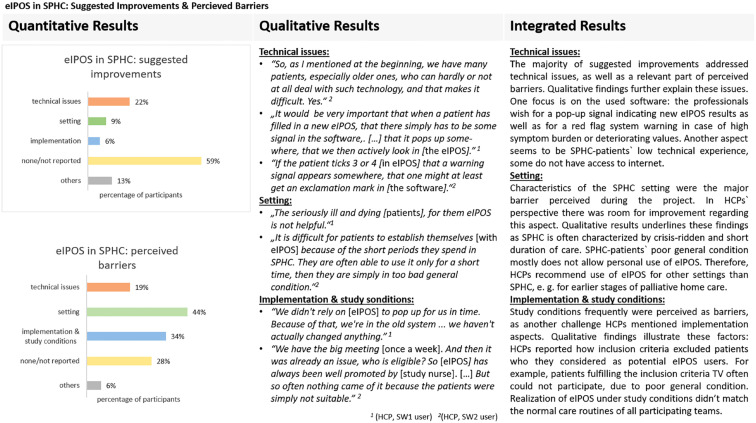
Joint display of the integrated data: eIPOS in SPHC - suggested improvements and perceived barriers.

More than half of the survey participants (*n* = 17, 53%) wished to use eIPOS after the project period. However, more than half of them (*n* = 10) linked this wish to necessary changes. Most of their comments addressed a change regarding technical issues (*n* = 5), three referred to the setting of SPHC and two related it to the implementation process. A detailed overview of the suggestions for improvement as well as of perceived barriers is provided in the joint display of the results from the focus groups and survey (see Figure 2).

Focus groups provided further results about eIPOS use. HCPs revealed the advantages using eIPOS in additional settings and populations of palliative home care (see Table 3). Support from relatives was mentioned as one possibility to enable eIPOS use for patients with high symptom burden or little technical practice.

**Table 3.** Additional qualitative results.

**Topic 1: Potentials regarding detecting and monitoring symptom burden** With those patients who were able to use eIPOS, HCPs identified the potential for clinical care routine. Symptoms and problems reported via eIPOS offer additional information compared with what has been documented by HCPs or what topics patients bring up in phone calls or face-to-face conversations, for example, about psychosocial issues. In addition, eIPOS results support organization and priority setting in clinical care routines.
• *“We didn’t have many patients that could [use eIPOS]. But for those, who did, I found it great as a supplement. I sit at the computer in the morning for team coordination. And seeing . . . that someone clicked a 3 or a 4, simply gave me a hint that we have to be active today”*. (HCP, SW2 user) • *“I always found it exciting to see what . . . the doctor writes down and what the patient directly submits [via eIPOS]. It is not always so completely identical. Or there is simply another aspect that has become visible”*. (HCP, SW2 user) • *“I believe that patients don’t usually say they are worried or that their family is worried in face-to-face conversations or on the telephone. . . . This often became more clear looking the eIPOS”*. (HCP, SW1 user) • *“It’s also good that you can enter free text. . . . Things are brought to the point there that . . . often cannot be addressed in conversation . . . But you can then go into it in the conversation, if it has already been mentioned”*. (HCP, SW1 user)
**Topic 2: Advantages of eIPOS in different settings and populations** HCPs emphasized the advantages of eIPOS use as a monitoring system for palliative care patients who are in intermitted or less close contact with their care team. As concrete settings, participants suggest eIPOS implementation in lower intensity levels of SPHC or for early integration palliative care patients in general palliative care and mention the possibility to include the general practitioner into eIPOS usage.
• *“Well, that would be very interesting, especially for those patients who are not currently being cared for [but have been cared for]. These are patients who … are more stable [so called ‘stillgelegte’ patients]. Especially for those patients, it would be very, very helpful to have a monitoring system, which actually gives signals: Now you have to contact them.”*. (HCP, SW1 user) • *It is sometimes a bit difficult to communicate when you say: “We will have to reduce the intensity of care”. Of course, you don’t say it like that. But if you then say: . . . “but we still have here, in any case, a tool [eIPOS] with which we can stay in close contact”. And so you would have the opportunity to offer them [the patients] something that also gives security”*. (HCP, SW1 user) • *“I have often thought to myself that it is a pity, that the patients we have in the so-called coordination [SPHC, less intensive level of care] would be more suitable for eIPOS. And of course it would be ideal if eIPOS results could be passed on directly to the GP”*. (HCP, SW2 user) • *“[eIPOS] for . . . early integration [of palliative care patients], that would be good. We often don’t really cover it with SPHC. We don’t have the capacity to take people so early. And I think it would be good if they were fitter”*. (HCP, SW1 user)

## Discussion

To our knowledge, this is one of the first studies to describe and explore the perspective of HCPs on the use of electronic PROMs in the clinical practice of SPHC. An important finding was that all HCPs had accessed the information provided via eIPOS and had discussed the patient statements submitted via eIPOS in their teams. Many HCPs felt that patients’ distress or symptoms and palliative care needs were sometimes better recognized thanks to eIPOS. The focus groups revealed differences between the two software programmes used in the four teams. However, the software itself was not the reason for whether the introduction of eIPOS was successful in everyday care. Rather, it was due to the specific organizational structure of the clinical practice and the patients cared for in the teams.

### HCPs’ perception on ePROM in (specialist) palliative home care

The results of the online survey highlighted that all HCPs reviewed the information provided through eIPOS. However, the online survey and focus groups showed that not all managed to check eIPOS regularly. Nevertheless, compared with other results, our findings show a relatively good compliance of HCPs using ePROM. Taarnhøj *et al.*^33^ found low compliance of physicians, but here ePROM software was not integrated into regular documentation software and the physicians had to log into a different software system. We found no difference between HCPs using the two different software systems. Compared with those who did not check eIPOS regularly, HCPs opening eIPOS often or always endorse future use of eIPOS in SPHC. Focus group results explain that those HCPs having more concrete experience with eIPOS use were more likely to report benefits or potentials. This is corroborated by previous findings that prove the motivation of HCPs as a main factor for PROM use.^27,34^ Furthermore, we identified perceived improvement in team communication as another factor influencing HCPs’ wish for further use of eIPOS.

### Effort of using eIPOS and integration of eIPOS into the daily care routine

While former studies described PROM use as time-consuming,^35,36^ our results regarding cost–benefit assessment were more divers. Most HCPs estimated the effort for the use of eIPOS as low. However, almost half of them stated that the benefit–effort ratio was not appropriate. As eIPOS realization differed between the two software systems, we tested association between perceived effort and documentation system. While we found no statistical significance here, documentation system proved to have a statistically significant influence on benefit-effort relation (in favour of SW1). In addition, the focus groups revealed that display of the submitted eIPOS in the patient record was an important aspect in the perception of HCPs. Nevertheless, our findings conformed the perceived effort of use to be an important factor that should be considered in the implementation of ePROM. For example, HCPs perceiving effort as low had a 16 times higher chance to interpret eIPOS values as helpful. This is consistent with former studies, showing that natural integration of PROMs’ feedback might reduce perceived effort.^27^ Furthermore, the results of the focus groups showed that the respective organizational structure and workflows in the teams are very important aspects for the successful integration of eIPOS. While the two teams using SW1 reported that they do not check documentation records on a daily basis, one team using SW2 stated the opposite, due to the completely different workflows. Therefore, there was the opportunity for them to check the transmitted eIPOS values regularly. The other team using SW2 worked with a closely integrated home care team. As a result, this team mainly supported patients in crisis situations who were not able to use eIPOS. Therefore, the team rarely had the opportunity to become proficient in its use. Grol and Wensing explained that implemented changes must also fit into the existing workflow.^20^ These results confirm previous studies that found the key factor for successful implementation of (e)PROM to be the smooth integration into organizational structures^34^ as well as the perceived effort.^27,29,33,35^

### Implications of eIPOS on care

Focusing on the setting of SPHC, our study underpins results of previous studies describing more benefits of PROM use, for example, regarding support of recognition of patients’ symptom burden and needs as well as improvement of communication with the patients and care.^6,9,13,37,38^ In our study, some respondents indicated that they noticed an improvement in the communication in the team. This result is also consistent with former findings.^39^ In the focus groups, HCPs identified a main benefit of eIPOS as tool to address and integrate psychosocial issues even more. This seems to be especially relevant, as participants stated that this aspect of holistic care tends to be neglected for the benefit of physical symptom burden in a crisis-ridden care situation. In a previous study, HCPs without experience with the ePROM stated in an interview that they doubted the suitability of this standardized assessment for psychosocial issues.^40^ These concerns have been eliminated by our findings. HCPs who noticed improvement in patients’ quality of life using eIPOS were more likely to see better identification in both patients’ burden and symptoms and palliative needs.

### Suggestions for improvement and perceived barriers

HCPs saw a need for improvement, especially in the technical implementation of eIPOS information in the patient record. In line with other studies, our data primarily support that the electronic implementation of PROM promotes effectiveness – assuming that the technical design meets the individual needs of the setting.^26,41–43^ However, adjusting technical issues does not help to overcome all setting specific barriers: our results confirm that many patients in SPHC might be too ill for (e)PROM use, as seen already in former research.^13,38^ Summarizing our results, most participants saw the potential of ePROM use in home-based palliative care – as far as it is technically easy to handle and can be easily integrated into daily work. An impressive and novel result is the connection between the different structures and processes of clinical practice in the participating teams and the HCPs’ perceptions of potentials regarding ePROM use in SPHC. HCPs suggested using eIPOS in home-based palliative care with less ill patients, involving general practitioners and family caregivers.

### Strengths and weaknesses

Due to the small sample of professionals and the close contact between the research team and the participating teams, age and gender were not indicated in the online survey to ensure anonymity. As a result, however, important confounding factors are missing from our analysis. This must be taken into account in the interpretation. Due to the COVID-19 pandemic and prolonged high workload in the SPHC teams, recruitment for the focus group discussion was challenging – the number of participants was low. However, the mixed-methods design compensated for this weakness. Mostly, integrating the data, qualitative findings provided explanation and deeper understanding of the quantitative results. Nevertheless, some contradictory results of the survey and focus groups could not be clarified with the available data. One reason could be that the survey was open to all HCPs who have experience with eIPOS, while participation in the focus groups was only possible for a few. As our results about professionals’ perspectives on ePROM use in palliative home care are based on a feasibility study of eIPOS in SPHC, some detailed findings are context and setting specific, for example, when addressing explicit organizational structures of SPHC or eIPOS-specific content. However, our general results about ePROM use in palliative home care can be also partly transferred to provide starting points for further research using alternative tools in different settings or populations of palliative care.

## Conclusion

Successful use of ePROM is crucially affected by the possibility of naturally integrating the system into the existing workflow. As the structure of SPHC in Germany is extremely diverse, we found varying HCPs’ perspectives on eIPOS. In some teams, structural and organizational issues mean that patients can only be cared for in SPHC in acute crisis and only shortly before they die. Introducing ePROM in this condition is disadvantageous; therefore, HCPs participating in our study recommend the use of ePROM in earlier stages of palliative home care or supported by relatives. On a policy level, equalization of SPHC framework conditions would be desirable.

## Supplemental Material

sj-pdf-1-pcr-10.1177_26323524231186827 – Supplemental material for Implementing ePROM in specialist palliative home care: the professionals’ perspective – a mixed-methods study Click here for additional data file. Supplemental material, sj-pdf-1-pcr-10.1177_26323524231186827 for Implementing ePROM in specialist palliative home care: the professionals’ perspective – a mixed-methods study by Isabel Burner-Fritsch, Stefanie Kolmhuber, Farina Hodiamont, Claudia Bausewein and Katerina Hriskova in Palliative Care and Social Practice

## Declarations

**Ethics approval and consent to participate:** The study was approved by the local research ethics committee at the Medical Faculty of Ludwig-Maximilians-University Munich (19-585). Written informed consent to participate was obtained from all participants.

**Consent for publication:** Informed consent for publication was provided by all participants.

**Availability of data and materials:** All data are available in German from the corresponding author on reasonable request.
