# Supplementary material for: Implementing ePROM in specialist palliative home care: the professionals’ perspective – a mixed-methods study
Source: Palliat Care Soc Pract. 2023 Aug 6;17:26323524231186827. doi: 10.1177/26323524231186827 (PMC10408331; doi:10.1177/26323524231186827)
Supplement: sj-pdf-1-pcr-10.1177_26323524231186827 – Supplemental material for Implementing ePROM in specialist palliative home care: the professionals’ perspective – a mixed-methods study [file sj-pdf-1-pcr-10.1177_26323524231186827.pdf]

Fragebogen

1. Bitte geben Sie an, inwieweit Sie den folgenden Aussagen in Bezug auf die patient*innenseitige Erfassung der Symptome und Palliativbedürfnisse eIPOS zustimmen:	nie	selten	gele-gentlich	oft	immer
Ich habe mir die Angaben der Patient*innen selbst angesehen.					
Wir haben die Angaben der Patient*innen im Team besprochen.					
Manche Belastungen der Patient*innen konnten durch die Erfassung mittels eIPOS besser erkannt werden.					
Manche Symptome und Palliativbedürfnisse der Patient*innen hätten wir ohne deren Angaben im eIPOS übersehen.					
Ich habe aufgrund der Angaben der Patient*innen die Versorgung in meinem Zuständigkeitsbereich angepasst.					
Die Angaben der Patient*innen waren nützlich für meine Arbeit.					
Die Erfassung der Symptome und Palliativbedürfnisse mittels eIPOS hat die Beziehung zu den Patient*innen beeinträchtigt.					
Ich habe die Angaben der Patient*innen als Anlass genutzt, mit den Patient*innen bestimmte Themen anzusprechen.					
Ich habe die Angaben der Patient*innen als Anlass genutzt, mit Kolleg*innen / im Team die Belastungen der Patient*innen anzusprechen.					

2. Haben Sie Auswirkungen des eIPOS auf die Versorgung oder die Behandlungsergebnisse wahrgenommen? Wo hat sich in Ihrer Wahrnehmung dadurch etwas verbessert? Wo etwas verschlechtert?	ver-schlechtert	eher ver-schlechtert	keine Veränderung	eher ver-bessert	verbes-sert
Behandlung **körperlicher Belastungen / Symptome**					
Behandlung **psychischer Belastungen**					
Beratung bei **sozialen Problemlagen**					
Begleitung bei **existentiellen Krisen**					
Begleitung bei **spirituellen / religiösen Anliegen**					
**Lebensqualität der Patient*innen**					
**Ärzt*innen-Patient*innen-Kommunikation**					
**Kommunikation über Belastungen der Patient*innen im Team**					

3. Denken Sie, dass der IPOS eine geeignete Grundlage für ein elektronisches Assessment bei SAPV-Patient*innen ist?
o Ja, weil:
o Nein, weil:

4. Inwieweit stimmen Sie Sie den folgenden Aussagen zur Integration der elektronischen Erfassung von Symptomen und Palliativbedürfnissen der Patient*innen in den Alltag zu:	Stimme überhaupt nicht zu	Stimme eher nicht zu	Stimme weder zu noch lehne ich ab	Stimme eher zu	Stimme voll zu
Insgesamt ist es uns gelungen, Routinen für die Verwendung der Angaben der Patient*innen zu entwickeln, die im Praxisalltag dauerhaft integrierbar sind.					
Der Aufwand für die Verwendung der Angaben der Patient*innen ist dem Nutzen angemessen.					
Die Darstellung des eIPOS in unserem Dokumentationssystem ermöglicht mir eine einfache Einbeziehung der Angaben der Patient*innen.					

5. Der Aufwand der Nutzung ist für die Patient*innen…

sehr gering	gering	hoch	sehr hoch

Der Aufwand der Nutzung ist für mich als Behandler*in…

sehr gering	gering	hoch	sehr hoch

6. Würden Sie eine Weiterführung der Erfassung der Symptome und Palliativbedürfnisse mittels eIPOS nach der Projektlaufzeit befürworten?

- o Nein, keine Weiterführung
- o Ja, ohne Veränderungen
- o Ja, aber mit folgenden Veränderungen:

7. Welche Vorschläge haben Sie insgesamt für Anpassungen oder Veränderungen der Erfassung der Symptome und Palliativbedürfnisse bei den Patient*innen in Ihrer Einrichtung?

8. Welche Hürden haben Sie während der Projektlaufzeit wahrgenommen?

9. Welches Dokumentationssystem verwenden Sie in Ihrem SAPV Team?

- o ISPC
- o PalliDoc
